# Dengue Infection in Children in Ratchaburi, Thailand: A Cohort Study. II. Clinical Manifestations

**DOI:** 10.1371/journal.pntd.0001520

**Published:** 2012-02-28

**Authors:** Chukiat Sirivichayakul, Kriengsak Limkittikul, Pornthep Chanthavanich, Vithaya Jiwariyavej, Watcharee Chokejindachai, Krisana Pengsaa, Saravudh Suvannadabba, Wut Dulyachai, G. William Letson, Arunee Sabchareon

**Affiliations:** 1 Department of Tropical Pediatrics, Faculty of Tropical Medicine, Mahidol University, Bangkok, Thailand; 2 Ratchaburi Hospital, Ministry of Public Health, Ratchaburi, Thailand; 3 Department of Disease Control, Ministry of Public Health, Nonthaburi, Thailand; 4 International Public Health Consultant, Denver, Colorado, United States of America; Pediatric Dengue Vaccine Initiative, United States of America

## Abstract

**Background:**

Dengue infection is one of the most important mosquito-borne diseases. More data regarding the disease burden and the prevalence of each clinical spectrum among symptomatic infections and the clinical manifestations are needed. This study aims to describe the incidence and clinical manifestations of symptomatic dengue infection in Thai children during 2006 through 2008.

**Study Design:**

This study is a school-based prospective open cohort study with a 9,448 person-year follow-up in children aged 3–14 years. Active surveillance for febrile illnesses was done in the studied subjects. Subjects who had febrile illness were asked to visit the study hospital for clinical and laboratory evaluation, treatment, and serological tests for dengue infection. The clinical data from medical records, diary cards, and data collection forms were collected and analyzed.

**Results:**

Dengue infections were the causes of 12.1% of febrile illnesses attending the hospital, including undifferentiated fever (UF) (49.8%), dengue fever (DF) (39.3%) and dengue hemorrhagic fever (DHF) (10.9%). Headache, anorexia, nausea/vomiting and myalgia were common symptoms occurring in more than half of the patients. The more severe dengue spectrum (i.e., DHF) had higher temperature, higher prevalence of nausea/vomiting, abdominal pain, rash, diarrhea, petechiae, hepatomegaly and lower platelet count. DHF cases also had significantly higher prevalence of anorexia, nausea/vomiting and abdominal pain during day 3–6 and diarrhea during day 4–6 of illness. The absence of nausea/vomiting, abdominal pain, diarrhea, petechiae, hepatomegaly and positive tourniquet test may predict non-DHF.

**Conclusion:**

Among symptomatic dengue infection, UF is most common followed by DF and DHF. Some clinical manifestations may be useful to predict the more severe disease (i.e., DHF). This study presents additional information in the clinical spectra of symptomatic dengue infection.

## Author Summary

Dengue infection is one of the most important diseases transmitted to human by mosquito bite. The disease may be mild or severe. This study reveals the occurrence and clinical features of diseases caused by dengue infection in a 3-year follow-up in school-children aged 3–14 years in Ratchaburi Province, Thailand using an active surveillance for the episodes of fever. Children who had fever were laboratory tested for the evidence of dengue infection and recorded for clinical features. It was found that most of dengue infected patients had headache, anorexia, nausea/vomiting, and muscle ache. About half of the patients had clinical symptoms that closely mimic other diseases, especially respiratory tract infection, and were incorrectly diagnosed by pediatricians. Only 11% of the patients had more a severe disease called “dengue hemorrhagic fever.” This severe disease may be predicted by the presence of anorexia, nausea/vomiting, and abdominal pain after the second day of illness. This study provides better understanding in this disease.

## Introduction

Dengue infection is one of the most important mosquito-borne viral diseases especially in tropical and subtropical regions of the world. World Health Organization (WHO) estimated that some 2.5 billion people – two-fifths of the world population are at risk from dengue and there may be 50 million dengue infections worldwide every year [1]. Its clinical spectrum ranges from asymptomatic infection to undifferentiated fever (UF), dengue fever (DF), and dengue hemorrhagic fever (DHF) [2]. In two studies, infected Thai children, ranging from 49 [3] to 87% [4], were asymptomatic. The available previous studies on the clinical manifestations in Thai children were a hospital-based study [5] which studied more severe disease, and a school-based study [3] which provided less detailed data on clinical manifestations. More data regarding the disease burden and the prevalence of each clinical spectrum (UF, DF, DHF) among symptomatic infections and the clinical manifestations are important for health care providers and policymakers to better understand the disease [6].

In 2005, a cohort epidemiological study of dengue infection in school-children aged 3–14 years was conducted in Ratchaburi Province, Thailand. There were 481 subjects recruited in 2005 and then 3056 subjects were enrolled in February, 2006. The initial plan was to conduct the study up to the end of 2008. In 2009, the surveillance for dengue epidemiology was extended. However, because many subjects withdrew from the study to participate a phase 2b dengue vaccine trial, the data on clinical manifestations was collected only up to the end of 2008. This article aims to describe the incidence and clinical manifestations of symptomatic dengue infection occurring over the 3-year study of this cohort, from 2006 to 2008.

## Methods

### Study Site

Ratchaburi Province is approximately 100 km west of Bangkok, the capital province of Thailand. It was ranked among the top ten provinces of Thailand for dengue incidence rate as reported to the Thai Ministry of Public Health. The study site is Muang District which is the capital district of the province. The population in this area is quite stable. There are low rates of migration and high ethnic homogeneity. The total population and number of children aged 5–14 years in Muang District was approximately 188,000 and 24,000, respectively. The data from Ratchaburi Provincial Health Office (unpublished) showed that the incidences of dengue diseases during 2006–2008 were 1.05, 1.15, and 0.24% in children aged 5–9 years, 10–14 years and total population, respectively. The principal medical facility in this area is Ratchaburi Hospital (RH), which is an 855-bed hospital providing tertiary medical care. The hospital has 90 pediatric beds and 12 pediatricians on staff.

### Study Population

School-children enrolled in 7 schools in Muang District were invited to participate in this cohort study. The inclusion criteria were healthy boys and girls aged 3–10 years at the time of recruitment, living in Muang District or nearby villages. Exclusion criteria included children suffering from serious or chronic severe diseases such as asthma, malignancy, hepatic, renal, cardiac disease or disease associated with insufficiency of immune system, and planning to move to another school within 48 months.

### Study Design

This study was a school-based prospective open cohort study. It is a part of the epidemiologic study of dengue infection in school children in Ratchaburi Province, Thailand. After explanation and obtaining informed consent, active surveillance for febrile episodes and laboratory confirmation for dengue infection were then conducted throughout the study period. Each year, new subjects were recruited to replace those who prematurely withdrew.

### Active Surveillance for Febrile Episode

All subjects/parent were provided with digital thermometers and were instructed to use the thermometer to measure and record the subjects' body temperature. The subjects were asked to visit RH if they had fever, defined as oral temperature ≥37.5°C. At the hospital, the study pediatrician performed physical examination and recorded the detail of illness. Tourniquet test was done by inflating a blood pressure cuff on the patient's upper arm to the point midway between the systolic and diastolic blood pressure for 5 minutes. The test was positive if 20 or more petechiae per square inch were observed. Blood samples for dengue diagnostic tests were collected from all febrile subjects irrespective of clinical diagnoses. Other laboratory tests such as complete blood count, liver function test, etc. were done when clinically indicated. Treatment was provided at the discretion of the pediatrician. In the subject who dengue infection was clinically diagnosed or suspected, or had no definite localizing sign of infection (e.g. purulent tonsillitis, otitis media, pneumonia, bacterial meningitis, etc.) and dengue infection could not be excluded, the pediatrician gave a diary card and asked the parent to record daily symptoms of the subject until recovery. The investigator checked and verified all diary cards on the day of convalescent blood drawing and asked the parent/caretaker to complete the missing data. If hospitalization was indicated, the pediatrician in charge recorded daily symptoms and physical findings, and laboratory results on a data collection form.

In addition to self-reporting febrile episode, an active surveillance for febrile episode based on subjects' school absence was also done. During school session, teachers reported school absentees every day and then project field coordinators called the subject's parent or visited the subject's home to verify the reason of school absence. If it was caused by fever, the subject was asked to visit RH as mentioned above. During school vacation and holidays, project field coordinators called the subjects' parents at least twice a week or performed home visit to ask whether the subject had febrile illness and remind the parent to take the subject to RH if he/she had febrile illness.

### Definition for Diagnosis

At RH, the pediatrician made clinical diagnosis without awareness of confirmatory laboratory test for dengue. Clinical diagnosis of DF, DHF and grading of DHF were made using WHO's criteria [2] after the subject recovered. Undifferentiated fever (UF) was defined if the subject did not meet the clinical criteria for DF (i.e. acute febrile illness with at least two of the following symptoms: headache, retro-orbital pain, myalgia, arthralgia, rash, hemorrhagic manifestations, leucopenia) or DHF (i.e. fever with hemorrhagic tendency, thrombocytopenia and evidence of plasma leakage) but was serologically proved to have dengue infection.

### Laboratory Test for Dengue Infection

Each subject was bled for acute and convalescent blood samples. The interval for the blood samplings was at least 7 days. The paired sera were tested for IgM and IgG against dengue using the enzyme-linked immunosorbent assay (ELISA) described by Innis et al [7]. Acute dengue infection was diagnosed if there was an IgM level higher than the cut-off value or there was a seroconversion of IgG level. IgM and IgG levels against Japanese encephalitis were also measured to exclude cross serologic reaction due to this infection. In addition to ELISA test which was use as primary diagnostic criterion for acute dengue infection, acute samples of the ELISA positive cases were further tested for dengue virus serotypes. In 2006, mosquito inoculation in *Toxorhynchites splendens* [8] was used for serotype identification. In 2007–8, the serotype diagnostic test used detection of viral RNA by a modified nested serotype-specific reverse-transcriptase polymerase chain reaction (RT-PCR) [9].

### Statistical Analysis

Although this study design was a school-based prospective open cohort study, most of the data in this article were cross-sectional data. Only symptoms were obtained from all days of illness from each subject and were analyzed longitudinally. The data were analyzed using SAS version 9.1.3 and Epi Info version 3.5.1. Frequency and median were used where appropriate to describe the data. Chi-square test or Fischer-exact test was used for comparing categorical variables and Kruskal-Wallis test or ANOVA-Scheffe test was used for comparing continuous variable as appropriate. The statistical level was considered significant when the p-value was <0.05.

### Ethical Approval

This study was approved by the Ethical Review Committee for Research in Human Subjects, Ministry of Public Health, Thailand. The informed consent signed by at least one parent or other legal guardian and assent form signed by the children if their age >7 years were obtained before recruiting the subject into the study.

## Results

A total of 9,448 (male 4,759, female 4,689) person-years were followed in the 3 years study period. There were 2,591 febrile episodes attended RH during this period. These represented 71% of the total febrile episodes occurring in the recruited subjects. Fifty-two percent of febrile episodes not attending RH were mild and were self-treated without medical attention. The remaining febrile patients who did not attend RH had attended either private clinics or hospitals. None of them were diagnosed as dengue infection because a study requirement was that a diagnosis of dengue outside RH would prompt a visit to RH. Our study follow-up team tracked all febrile patients to a conclusion and prompted all with a dengue diagnosis to visit RH. Among those who attended RH, 313 febrile episodes (12.1%) were due to dengue infections as diagnosed by ELISA test, including 41(13.1%) primary antibody responses and 272 (86.9%) secondary antibody responses. Dengue serotypes were identified by either mosquito inoculation or RT-PCR in 259 cases (82.7%) including 115 (36.7%), 80 (25.6%), 41 (13.1%), and 23 (7.3%) cases of DEN1, 2, 3, and 4, respectively. There were 156 cases (49.8%) of UF, 123 cases (39.3%) of DF, and 34 cases (10.9%) of DHF including 22 cases (7.0%), 5 cases (1.6%), and 7 cases (2.2%) of DHF grade 1, 2 and 3, respectively. Among 41 cases with primary antibody responses, 26 cases had UF (63.4%), 14 cases had DF (34.2%) and 1 case had DHF grade 1 (2.4%). There was no significant association among infecting dengue serotypes and types of antibody response (data not shown).

The detailed data on clinical manifestations (i.e. daily symptoms until recovery) of 71 UF cases were not obtained. Therefore only 85 UF cases are used for analysis. The clinical diagnosis of these 71 and 85 UF cases are shown in table 1. Significantly more cases in the group whose data were obtained were diagnosed as acute febrile illness/suspected viral infection (p<0.001). However, there was no statistically significant difference in other clinical diagnosis between groups. There was also no statistically significant difference by age and gender [8.9 (SD 2.2) *vs* 9.2 (SD 2.2) years and 46.5% *vs* 61.2% male gender, respectively] although the male proportions were rather skewed.

**Table 1:** Clinical diagnosis of UF cases.

Clinical diagnosis	Cases with missing data (%)(n = 71)	Cases used in analysis (%) (n = 85)
Acute febrile illness/Suspected viral infection^a^	4 (5.6)	26 (30.6)
Rhinitis/Common cold/Unspecified upper respiratory tract infection	19 (26.8)	14 (16.5)
Influenza	3 (4.2)	3 (3.5)
Rhinopharyngitis/Pharyngitis/Tonsillitis	37 (52.1)	35 (41.2)
Bronchitis	3 (4.2)	1 (1.2)
Sinusitis	0	1 (1.2)
Gastritis/Gastroenteritis	2 (2.8)	4 (4.7)
Viral exanthem	1 (1.4)	1 (1.2)
Rubella	1 (1.4)	0
Bee sting	1 (1.4)	0

a Statistically significant difference.

The median age (interquatile range [IQR]) and percentage of males in the cases who had UF, DF and DHF were 9.3 (3.3), 9.6 (3.3), 10.0 (3.3) years, and 55.1, 55.3, 58.8 percent, respectively. There was no statistically significant difference in the age and gender among different disease spectra.

The symptoms of overall dengue infection and each specific disease spectrum (UF, DF, DHF) are presented in table 2. Headache, anorexia, nausea/vomiting and myalgia were common symptoms occurring in more than half of the patients. It was found that nausea/vomiting, abdominal pain, rash, diarrhea and petechiae were statistically more common in DHF compared to DF and UF. Table 3 shows symptoms and disease severity from different dengue serotypes. DEN4 was found to cause only UF, DF and DHF grade 1 and seemed to have less headache, anorexia, nausea/vomiting and rash compared to other DEN serotypes. However, these differences are not statistically significant.

**Table 2:** Symptoms of dengue infection [n (%)].

Symptom	UF(n = 85)	DF(n = 123)	DHF(n = 34)	Total(n = 242)	p-value
Headache	76 (89.4)	107 (87.0)	25 (73.5)	208 (86.0)	0.07
Anorexia	69 (81.2)	96 (78.0)	31 (91.2)	196 (81.0)	0.22
Nausea/Vomiting	58 (68.2)	95 (77.2)	32 (94.1)	185 (76.4)	0.01^a^
Myalgia	41 (48.2)	65 (52.8)	21 (61.8)	127 (52.5)	0.41
Abdominal pain	32 (37.6)	59 (48.0)	25 (73.5)	116 (47.9)	0.002^a^
Rhinorrhea	37 (43.5)	39 (31.7)	12 (35.3)	88 (36.4)	0.22
Retro-orbital pain	32 (37.6)	35 (28.5)	13 (38.2)	80 (33.1)	0.30
Rash (convalescent)	6 (7.1)	53 (43.1)	18 (52.9)	77 (31.8)	<0.0001^b^
Drowsiness/Lethargy	27 (31.8)	23 (18.7)	6 (17.6)	56 (23.1)	0.06
Diarrhea	17 (20.0)	21 (17.1)	17 (50.0)	55 (22.7)	<0.001^a^
Arthralgia	23 (27.1)	17 (13.8)	6 (17.6)	46 (19.0)	0.06
Petechiae	6 (7.1)	25 (20.3)	15 (44.1)	46 (19.0)	<0.0001^c^
Epistaxis	4 (4.7)	13 (10.6)	5 (14.7)	22 (9.1)	0.17
Gum bleeding	0	5 (4.1)	1 (2.9)	6 (2.5)	NA
Oliguria	0	0	4 (11.8)	4 (1.7)	NA
Hematemesis	0	0	1 (2.9)	1 (0.4)	NA
Melena	0	0	1 (2.9)	1 (0.4)	NA

a significantly more common in DHF compared to DF and UF, but no difference between UF and DF.

b significantly less common in UF compared to DF and DHF, but no difference between DF and DHF.

c significantly more common in DHF compared to DF and UF, and more common in DF compared to UF.

**Table 3:** Symptoms and disease severity from different dengue serotypes [n (%)].

Symptom	DEN1(n = 91)	DEN2(n = 59)	DEN3(n = 32)	DEN4(n = 17)	p-value
Headache	85 (93.4)	57 (96.6)	31 (96.8)	14 (82.4)	0.15
Anorexia	77 (84.6)	47 (79.7)	27 (84.4)	11 (64.7)	0.26
Nausea/Vomiting	75 (82.4)	45 (76.3)	23 (71.9)	11 (64.7)	0.32
Myalgia	58 (63.7)	44 (74.6)	19 (59.4)	11 (64.7)	0.43
Abdominal pain	66 (72.5)	32 (54.2)	24 (75.0)	9 (52.9)	0.05
Rhinorrhea	47 (51.6)	30 (50.8)	16 (50.0)	5 (29.4)	0.40
Retro-orbital pain	56 (61.5)	32 (54.2)	19 (59.4)	11 (64.7)	0.80
Rash (convalescent)	29 (31.9)	14 (23.7)	12 (37.5)	2 (11.8)	0.19
Drowsiness/Lethargy	18 (19.8)	16 (27.1)	12 (37.5)	5 (29.4)	0.24
Diarrhea	21 (23.1)	12 (20.3)	11 (34.4)	6 (35.3)	0.35
Arthralgia	36 (39.6)	19 (32.2)	11 (34.4)	7 (41.2)	0.79
Petechiae	23 (25.3)	10 (16.9)	9 (28.1)	4 (23.5)	0.58
Epistaxis	9 (9.9)	3 (5.1)	4 (12.5)	2 (11.8)	0.61
Gum bleeding	2 (2.2)	0	2 (6.3)	0	NA
Hematemesis	2 (2.2)	2 (3.4)	2 (6.3)	1 (5.9)	0.69
Melena	0	0	1 (3.1)	0	NA
Disease spectra					
UF	58 (50.4)	43 (53.8)	14 (34.1)	11 (47.8)	
DF	48 (41.7)	27 (33.8)	21 (51.2)	10 (43.5)	
DHF grade 1	5 (4.3)	7 (8.8)	3 (7.3)	2 (8.7)	
DHF grade 2	1 (0.9)	1 (1.3)	2 (4.9)	0	
DHF grade 3	3 (2.6)	2 (2.5)	1 (2.4)	0	

Regarding fever, the median (IQR) value of the peak temperature and duration of fever in UF, DF, and DHF were 38.4 (1.6), 39.0 (1.5), and 39.0 (1.7) degree Celsius and 5 (3), 6 (2), and 6 (1) days, respectively. It was found that UF had both lower peak temperature and shorter duration of fever compared to DF and DHF (p<0.001).

**Figure 1:**
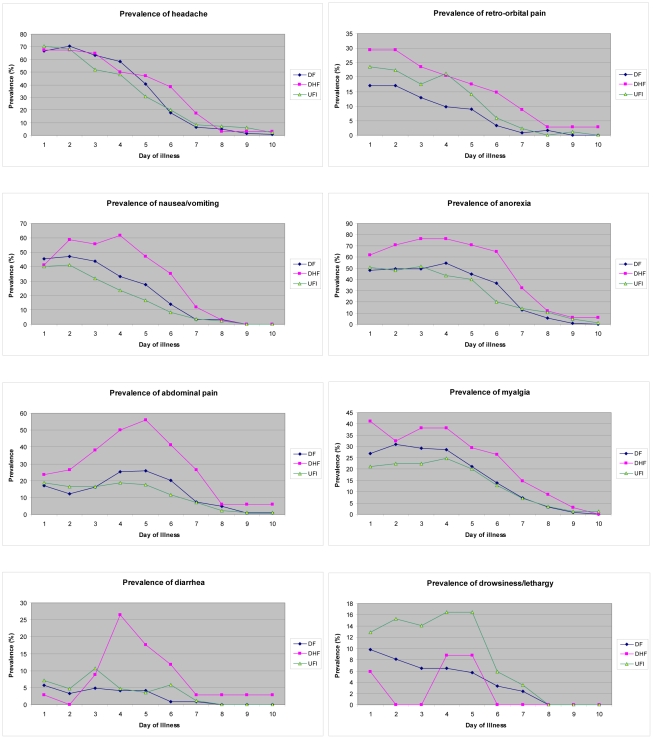
The prevalence of common symptoms in dengue infection by the day of illness.

The prevalence of common symptoms in each day of illness is shown in figure 1. It was noted that the prevalence of most of the symptoms was highest during the first 2 days of illness and then slowly declined. The exceptions are for anorexia, nausea/vomiting, abdominal pain and diarrhea that tended to increase in prevalence in DHF during day 3–5 of illness. It was also revealed that most symptoms were more common in DHF and, specifically, DHF cases had significantly higher prevalence of anorexia, nausea/vomiting and abdominal pain during day 3–6 and diarrhea during day 4–6 of illness (Chi-square test; p<0.05). Conversely, drowsiness/lethargy was significantly more common in UF during day 2–5 of illness.

It was also revealed that a higher proportion of DHF had prolonged (3 or more days) anorexia, nausea/vomiting, abdominal pain, retro-orbital pain and petechiae. In contrast, a higher proportion of UF had prolonged drowsiness/lethargy. It is worth noting that very few cases had prolonged hemorrhagic manifestation (Table 4).

**Table 4:** The number of dengue infected patients who had prolonged symptom (for 3 or more days) [n (%)].

Symptom	UF(n = 85)	DF(n = 123)	DHF(n = 34)	Total(n = 242)	p-value
Headache	50 (58.8)	83 (67.5)	22 (64.7)	155 (64.0)	0.44
Anorexia	43 (50.6)	70 (56.9)	27 (79.4)	140 (57.9)	0.02^a^
Nausea/Vomiting	24 (28.2)	52 (42.3)	19 (55.9)	95 (39.3)	0.01^b^
Myalgia	22 (25.9)	38 (30.9)	10 (29.4)	70 (28.9)	0.73
Abdominal pain	18 (21.2)	26 (21.1)	16 (47.1)	60 (24.8)	<0.01^a^
Rhinorrhea	19 (22.4)	13 (10.6)	6 (17.6)	38 (15.7)	0.07
Retro-orbital pain	19 (22.4)	12 (9.8)	8 (23.5)	39 (16.1)	0.02^d^
Rash (convalescent)	1 (1.2)	0	5 (14.7)	6 (2.5)	NA
Drowsiness/Lethargy	14 (16.5)	7 (5.7)	0	21 (8.7)	<0.01^c^
Diarrhea	3 (3.5)	2 (1.6)	2 (5.9)	7 (2.9)	0.39
Arthralgia	0	0	0	0	NA
Petechiae	2 (2.4)	11 (8.9)	7 (20.6)	20 (8.3)	<0.01
Epistaxis	0	5 (4.1)	0	5 (2.1)	0.24
Gum bleeding	0	1 (0.8)	0	1 (0.4)	NA
Oliguria	0	0	0	0	NA
Hematemesis	0	0	0	0	NA
Melena	0	0	0	0	NA

NA = not applicable.

a significantly more common in DHF compared to DF and UF, but no difference between DF and UF.

b significantly less common in UF compared to DF and DHF, but no difference between DF and DHF.

c significantly more common in UF compared to DF and DHF, but no difference between DF and DHF.

d significantly less common in DF compared to UF and DHF, but no difference between UF and DHF.

Table 5 shows physical findings in dengue infection. Positive tourniquet test was found in 72% and flushed face was found in approximately half of the dengue infected patients. Hepatomegaly was found in approximately 40% of DHF. This proportion was significantly higher than that found in UF and DF. Clinical jaundice was found only in one DHF (2.9%). Table 6 shows the occurrence of selected clinical manifestations and their predictive value for DHF among symptomatic dengue infected cases. It was found that all of these clinical manifestations had low positive predictive value but high negative predictive value for DHF.

**Table 5:** Physical findings in dengue infection [n/number observed (%)].

Finding	UF	DF	DHF	Total	p-value
Flushed face	33/83(39.8)	61/122(50.0)	21/33(63.6)	115/238(48.3)	0.06
Positive tourniquet test	21/54(38.9)	91/107(85.0)	27/31(87.1)	139/192(72.4)	<0.001^a^
Hepatomegaly	4/79(5.1)	21/121(17.4)	14/34(41.2)	39/234(16.7)	0.001^b^
Cervical lymphadenopathy	7/76(9.2)	12/117(10.3)	6/33(18.2)	25/226(11.1)	0.36

a significantly less common in UF compared to DF and DHF, but no difference between DF and DHF.

b significantly more common in DHF compared to DF and UF, and more common in DF compared to UF.

**Table 6:** Occurrence of selected clinical manifestations [n/total] and their predictive value (%) for DHF among dengue infected cases.

Clinical Manifestation	DHF	Non-DHF(UF+DF)	Positive Predictive Value	Negative Predictive Value
Nausea/Vomiting	32/34	153/208	17.3	96.5
Abdominal pain	25/34	91/208	21.6	92.9
Diarrhea	17/34	38/208	30.9	90.9
Petechiae	15/34	31/208	32.6	90.3
Anorexia >3 days	27/34	113/208	19.3	93.1
Nausea/Vomiting >3 days	19/34	76/208	20.0	89.8
Abdominal pain >3 days	16/34	44/208	26.7	90.1
Petechiae >3 days	7/34	13/208	35.0	87.8
Positive tourniquet test	27/31	112/161	19.4	92.5
Hepatomegaly	14/34	25/200	35.9	89.7

**Figure 2:**
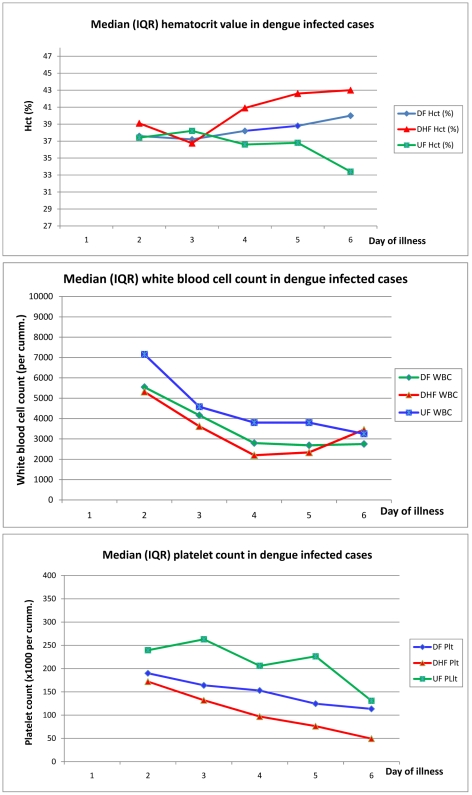
Hematocrit, peripheral white blood cell and platelet count in dengue infected patients.

Complete blood count were done in 43, 11, 8, 2, 2, 2 UF and in 113, 69, 32, 20, 7, 4 DF, and 32, 19, 12, 8, 2, 2 DHF cases on day 1, 2, 3, 4, 5, 6 of illness, respectively. Figure 2 shows the median value of hematocrit, peripheral white blood cell and platelet count in these dengue-infected patients. It was revealed that DHF cases had higher median hematocrit during the later days of illness and the median hematocrit in DHF was significantly higher than DF and UF during day 4–6 of illness. All of the 3 spectra of dengue infection had lower median peripheral white blood cell (WBC) count during the later days of illness and cases with UF had significantly higher median WBC count compared to DF and DHF. All spectra of dengue also had lower median peripheral platelet count during the later days of illness, although the median value in DF and UF did not meet the WHO's criterion for thrombocytopenia, i.e. lower than 100,000/mm^3^. Moreover, the level of platelet count showed reverse correlation with the disease severity, i.e. highest in UF and lowest in DHF. The median platelet count in DHF was significantly lower than the other 2 groups during day 3–6 of illness, while DF had significantly lower median platelet count compared to UF only during day 3–5 of illness.

## Discussion

This was a prospective cohort study and the data represent mild to severe dengue illnesses. The data have been prospectively collected, diagnoses of dengue were serologically proven and the observations were representative of a defined pre-illness population. Because confirmatory laboratory results were available after convalescence, the pediatricians made diagnosis solely based on WHO's criteria [2] without bias from the etiologic investigation results. This provides us the opportunity to make the diagnosis of UF; one clinical spectrum of dengue infection that has been mentioned but poorly defined [2] and describe its clinical manifestations.

Although there have been many published data on clinical manifestations of dengue, this study addresses the large proportion of clinical dengue that is often unrecognized by physicians. This provides useful information for policy-makers in endemic countries because the impact of dengue has likely been underestimated, based on the more standard clinical definitions. Recently, prospective cohort studies on the dengue incidence in Cambodia and Nicaragua using community-based enhanced passive surveillance were published [10]–[11]. Similar to this study, they reported a high incidence of dengue infection in these endemic areas. However, the detailed clinical manifestations of dengue-infected cases were not mentioned.

This study describes the prevalence of each clinical symptom day by day starting from the onset of illness, which has never been reported in outpatient setting. It was found that some clinical symptoms, for example, nausea/vomiting, diarrhea, abdominal pain are significantly more common in DHF compared with non-DHF. Although these clinical manifestations have low positive predictive value for DHF that might be due to much higher incidence of non-DHF cases (Table 6), they have quite high negative predictive value. The absence of these clinical manifestations suggests that the patient should have non-DHF while the presence of them should alert the physician to be watchful for DHF.

The high incidence of UF cases that did not meet the clinical criteria for DF or DHF was not anticipated in the study design. In a large proportion of laboratory confirmed cases of UF dengue (71 or 45.5%), the clinical manifestations of these cases were not obtained because the doctors on duty did not think that the patients might have dengue infection and did not ask the patients to record their symptoms. Moreover, many patients especially those who had UF and DF, had mild illness. They visited the out-patient department only once during the febrile phase and therefore many data on physical findings and laboratory investigations were not obtained. These may be sources of bias resulting in overestimating or underestimating the clinical manifestations of UF dengue infection and is a weakness in this study. Nevertheless, the largely similar clinical diagnoses and demographics between the UF patients whose clinical data were available and the patients whose data were missed implied that the available clinical data are generally representative of UF cases. The difference in the diagnosis of acute febrile illness/suspected viral infection is probably reflective of an overestimate of clinical severity among UF patients since the patients whose clinical data was collected were more likely to have a clinical presentation common to many viral illnesses. This would suggest that UF may be even milder than we report here. Nonetheless, these data demonstrate several interesting aspects of mild dengue infection, which we classified as UF, that do not meet case criteria for DF.

There may be argument that these UF cases may be asymptomatic dengue with co-infection by another agent. Although this is possible, we estimated that these events might not be very common because the time these cases presented was not the outbreak period of other infection. In addition, the high degree of similarity in many clinical symptoms in UF patients compared to DF and DHF patients (Table 2), suggests that most of the UF patients really had symptomatic dengue disease.

The prevalence of overall clinical manifestations of dengue infection presented in this report is within the range of that previously reported, including much older reports (Table 7). However, it is worth emphasizing that the data from each study presented in table 7 are not comparable due to different study design, population (age and ethnicity), settings (i.e. community *vs.* hospital-based and in- *vs.* out-patient setting), which all could lead to biases in the clinical manifestations.

**Table 7:** The prevalence (%) of clinical manifestation of dengue diseases from previous studies.

Clinical manifestation	Hammond et al. [12]	Kalayanarooj et al.[5]	Nimmannitya et al. [13]	Halstead et al. [14]	Endy et al. [3]	Wichmann et al. [15]
Patient's characteristic	In-patientchildren	In- patientchildren	In-patientchildren	Out-patientchildren	In- and out- patientchildren	In-patient adults and children
Headache	78	77	45	20	64	15
Anorexia	59	85	ND	8	25	ND
Nausea	ND	68	ND	ND	19	57
Vomiting	ND	70	58	24	24	59
Myalgia/Arthralgia	66/62	ND/ND	12^a^	6/1	23/15	8^a^
Abdominal pain	62	34	50	7	17	ND
Rhinorrhea	ND	ND	13	ND	35	ND
Retro-orbital pain	59	ND	ND	ND	ND	ND
Rash (convalescent)	40	ND	10	ND	5	ND
Drowsiness/Lethargy	ND	ND	ND	ND	32	ND
Diarrhea	17	ND	6	3	4	ND
Petechiae	38	ND	47	ND	ND	ND
Epistaxis	21	ND	19	3	ND	ND
Gum bleeding	5	ND	2	ND	ND	ND
Hematemesis	6	ND	ND	ND	ND	ND
Melena	4	ND	12	ND	ND	ND
Positive tourniquet test	42	43	84	ND	ND	ND
Hepatomegaly	22	ND	ND	ND	ND	40

ND = no data.

a include both myalgia and arthralgia.

Data from inpatients (usually DHF) are more reliable, have more detail, and are therefore less biased when compared to outpatients (usually DF and UF). However, among the subjects who were given diary cards, the 100% return rate of diary cards and verification of the diary card data with parent/caretaker on the day of convalescent blood drawing would decrease the bias in outpatient data. Because of the lower proportion of viral syndrome presented in the UF cases without diary card, we feel there was a conservative bias for the UF cases, i.e. the illness profile was likely even milder in this UF group than seen in the data we were able to collect.

It is not surprising that the more severe disease spectrum (i.e. DHF) had higher prevalence and longer duration of symptoms. The exceptions were found in headache and drowsiness/lethargy. We do not have an explanation for this finding. Further studies are needed to clarify this issue. It is interesting that rhinorrhea was commonly found in this study. Whether this symptom is one manifestation of dengue infection or is caused by co-incidental respiratory infection is still unclear. However, it has been suggested that dengue infection should be included in the differential diagnosis of acute infection of the upper respiratory tract [14]. Facial flushing was also commonly found. This distinct clinical feature was used as an enrolment criterion for suspected dengue infection in one study [5]. It is worth noting that some clinical manifestations (e.g. rash, positive tourniquet test) were considered as more specific to DF/DHF and are included in clinical diagnostic criteria for DF. This can explain why these clinical manifestations were more common in DF/DHF. The same reason is also applicable to the finding that hepatomegaly was more common in DHF.

Mild dengue infection is quite similar to many other infections in that the prevalence of most of the symptoms was highest during the first few days of illness and then slowly declined. On the other hand, in severe dengue infection (i.e. DHF), the prevalence of anorexia, nausea/vomiting, abdominal pain and diarrhea during day 3–5 of illness was found to increase instead of decrease and it will indicate the risk for more severe disease.

The presence of diarrhea in dengue infection has been mentioned in many previous studies [3], [12]–[14]. However, there has been no study on its mechanism. This study, to the best of our knowledge, is the first study suggesting that diarrhea may be a predictor for DHF. Its highest prevalence just before the stage of shock suggests that it may be related to plasma leakage that may cause malabsorption. Further study to clearly define the association between diarrhea and DHF and its mechanism is warranted.

This study shows that a decrease in peripheral platelet count occurs not only in DHF, but also in DF and UF, with a respectively less degree. It confirms that the cut off level for platelet count of 100,000/mm^3^ is appropriate for differentiation between DHF and non-DHF. Nevertheless, complete blood counts were done selectively by clinicians based on clinical need and may not be accurately extrapolated to all patients with UF and DF. In addition, there were variations around this median value and may reduce predictive value for individual patient.

Infecting dengue virus serotype has been postulated as a risk factor for severe disease. For example, DEN2 and 3 have been shown to be associated with more severe disease [16], [17] and DEN4 with mild disease [18]. This study also found that DEN4 did not cause severe disease. However, we could not show the statistically significant association between specific virus serotypes and disease severity.

While the WHO's new guidelines for dengue diagnosis [19] classifies dengue infection into severe and non-severe infection and has a potential for being of practical use in the clinicians' decision and management, this new guideline as well as the earlier WHO classification [2] may easily miss the UF cases because the clinician will not diagnose dengue when seeing patients who present in the fashion of the UF group described here, and therefore confirmatory diagnostic tests for dengue will not be done. These UF cases may have important role in spreading of infection and may explain the failure of disease control in some endemic area where vector control is implemented only around the house of index DF and DHF, but not UF patients.

It is stated in the WHO's new guideline for dengue diagnosis [19] that the presence of abdominal pain or tenderness, persistent vomiting, clinical fluid accumulation, mucosal bleed, lethargy, restlessness, liver enlargement are clinical warning signs of severe dengue disease. The finding in this study that DHF cases had higher prevalence of nausea/vomiting and abdominal pain supports this guideline. On the other hand, we found that the presence of lethargy or drowsiness in the first few days of illness may not necessarily indicate severe dengue infection. The significance of drowsiness/lethargy needs to be further defined.

In conclusion, this study describes the clinical manifestations of all spectra of symptomatic dengue infection as well as some possible early clinical predictors for DHF. It also reveals the epidemiological importance of UF as the most common spectrum of symptomatic dengue infection. This study presents additional information in the clinical spectra of symptomatic dengue infection.

## Supporting Information

Checklist S1 SEARD checklist.
(DOC)
